# Dual-Warhead Conjugate Based on Fibroblast Growth Factor 2 Dimer Loaded with α-Amanitin and Monomethyl Auristatin E Exhibits Superior Cytotoxicity towards Cancer Cells Overproducing Fibroblast Growth Factor Receptor 1

**DOI:** 10.3390/ijms241210143

**Published:** 2023-06-14

**Authors:** Daria Nawrocka, Mateusz Adam Krzyscik, Katarzyna Dominika Sluzalska, Jacek Otlewski

**Affiliations:** Department of Protein Engineering, Faculty of Biotechnology, University of Wroclaw, Joliot-Curie 14a, 50-383 Wroclaw, Poland; daria.nawrocka@uwr.edu.pl (D.N.); mkrzysc1@jhu.edu (M.A.K.); katarzyna.sluzalska@uwr.edu.pl (K.D.S.)

**Keywords:** fibroblast growth factor 2 (FGF2), dimerization, MMAE, α-amanitin, protein-drug conjugates, sortase A, SnoopLigase, targeted cancer therapy

## Abstract

Targeting fibroblast growth factor receptor 1 (FGFR1) is a promising therapeutic strategy for various cancers associated with alterations in the FGFR1 gene. In this study, we developed a highly cytotoxic bioconjugate based on fibroblast growth factor 2 (FGF2), which is a natural ligand of this receptor, and two potent cytotoxic drugs—α-amanitin and monomethyl auristatin E—with completely independent mechanistic modes of action. Utilizing recombinant DNA technology, we produced an FGF2 N- to C-end dimer that exhibited superior internalization capacity in FGFR1-positive cells. The drugs were site-specifically attached to the targeting protein using SnoopLigase- and evolved sortase A-mediated ligations. The resulting dimeric dual-warhead conjugate selectively binds to the FGFR1 and utilizes receptor-mediated endocytosis to enter the cells. Moreover, our results demonstrate that the developed conjugate exhibits about 10-fold higher cytotoxic potency against FGFR1-positive cell lines than an equimolar mixture of single-warhead conjugates. The diversified mode of action of the dual-warhead conjugate may help to overcome the potential acquired resistance of FGFR1-overproducing cancer cells to single cytotoxic drugs.

## 1. Introduction

Alterations in the fibroblast growth factor receptor 1 (FGFR1) have been linked to the development and progression of several types of cancer, including breast, lung, gastric, bladder, and ovarian cancers, and is associated with poor prognosis and resistance to chemotherapy [1,2,3]. FGFR1 signaling is involved in multiple pathways that promote cancer cell proliferation, survival, angiogenesis, and tumor progression, making FGFR1 an attractive therapeutic target in cancer treatment [4,5].

FGFR-targeted therapies have emerged as a promising approach to inhibit FGFR1 signaling and selectively target cancer cells with FGFR1 overexpression. To date, several strategies have been developed, including small molecule inhibitors [6] and monoclonal antibodies [7]. Small molecule FGFR inhibitors are designed to block the ATP binding site of FGFR1, preventing ATP-dependent phosphorylation and downstream signaling. These inhibitors exhibit varying degrees of selectivity against FGFR isoforms and have shown promising results in preclinical and clinical studies. Examples of small-molecule FGFR inhibitors include erdafitinib, pemigatinib, and infigratinib, among others [8,9,10,11,12,13,14,15]. Monoclonal antibodies targeting FGFR1 aim to block FGFR1 activation by inhibiting ligand binding or receptor dimerization. These antibodies can interfere with FGFR1 signaling and trigger immune-mediated mechanisms of action, such as antibody-dependent cellular cytotoxicity (ADCC) and complement-dependent cytotoxicity (CDC). Clinical trials evaluating FGFR1-targeted monoclonal antibodies, such as bemarituzumab and futibatinib, are underway in various cancer types [16,17,18,19,20,21,22,23].

Several preclinical and clinical studies have shown promising outcomes for treatments targeting FGFR1, demonstrating encouraging response rates and manageable toxicity profiles. However, similar to other targeted therapies, resistance to FGFR1 inhibitors can emerge, either through mutations in the target protein or activation of bypass pathways [24,25]. Thus, to overcome the resistance, it is necessary to utilize combination therapies or sequential treatment with different FGFR1 inhibitors.

Previously, we demonstrated the efficacy of fibroblast growth factor 1 (FGF1) and 2 (FGF2) as a targeting molecules for FGFR1-overproducing cancer cells. These natural FGFR1 ligands, when conjugated with a potent anticancer drug, such as monomethyl auristatin E (MMAE), exhibited a highly selective cytotoxic effect against cancer cells [26,27,28,29]. In addition, the cytotoxicity of FGF2-based conjugates was improved by adding two mechanistically distinct warheads [30,31]. Furthermore, we engineered FGF2 dimers that demonstrated remarkable stability and exceptional internalization efficiency into cells overproducing FGFR1 [32]. Achieving efficient internalization of bioconjugates is critical for developing delivery systems for highly cytotoxic drugs. Here, we expand on the FGF-based targeted anti-cancer cytotoxic drug approach by applying the FGF2 dimer as a targeting molecule and through conjugation of two cytotoxic agents with independent modes of action: the antimitotic agent MMAE and the RNA polymerase II and III inhibitor α-amanitin (αAMTN) using two enzymatic ligations. Application of the FGF2 dimer extended by N- and C-terminal peptide tags recognized, respectively, by SnoopLigase and sortase A as a drug delivery vehicle enabled site-specific conjugation of each cytotoxic payload and allowed for precise control over each conjugation step, allowing stoichiometrically defined conjugates to be obtained.

Our results show that the developed conjugate efficiently targets FGFR1-positive cancer cells, leading to excellent and selective toxicity. Compared with single-drug monomeric conjugates, the dimeric FGF2 dual warhead conjugate kills cancer cells more efficiently and has the potential to limit the ability of cancer cells to develop resistance to cytotoxic drugs.

## 2. Results and Discussion

### 2.1. Engineering the Dimeric Dual-Warhead FGF2 Conjugate

#### 2.1.1. Design Principle of the FGF2 Dimer (dFGF2_V1V2_)

Our recent report highlights the successful synthesis of FGF2 dimers with variations in both topology and length of the linker used for dimerization [32]. The engineered dimers remained functional in terms of FGFR downstream signaling activation and demonstrated remarkable stability. One of the major findings from the previous study was that the arrangement of FGF2 molecules within the dimer is a crucial factor in facilitating effective internalization into cells that overproduce FGFR1. Achieving efficient internalization of bioconjugates is critical in the development of delivery systems for highly cytotoxic drugs. This allows for the specific release of the active form of the cytotoxic compound exclusively within the targeted cells [33].

**Figure 1:**
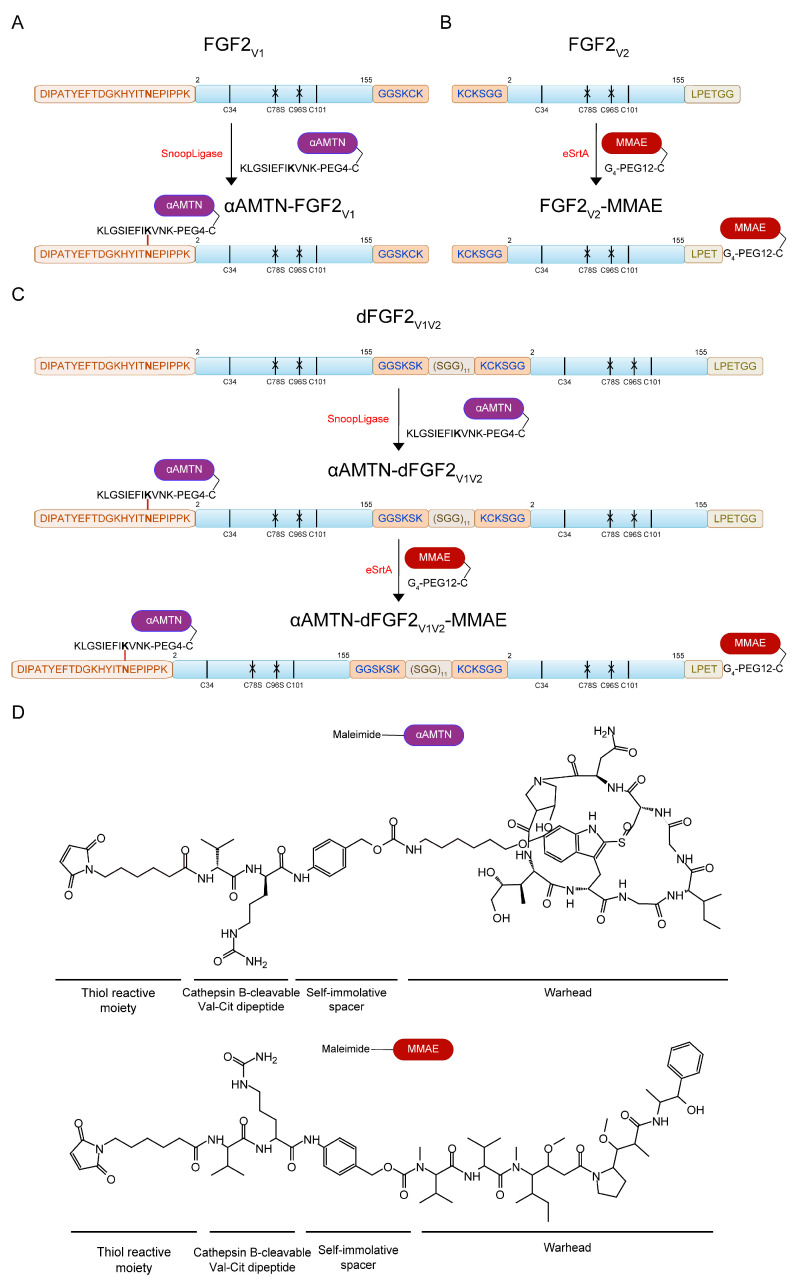
Schematic representation of the conjugation procedure. (**A**) Conjugation of α-amanitin to FGF2_V1_ through SnoopLigase-mediated ligation. SnoopLigase promotes an isopeptide bond formation between a reactive asparagine located on the peptide tag attached to the N-terminus of FGF2 and a lysine residue in the sequence of the second peptide that is recognized by the enzyme, coupled with α-amanitin. (**B**) Conjugation of monomethyl auristatin E to the FGF2_V2_ using evolved sortase A. Sortase A recognizes LPETGG motif at the C-terminus of FGF2 and cleaves the threonine–glycine amide bond, then catalyzes the formation of a new peptide bond between the threonine residue and the amino group of a glycine residue on an incoming oligoglycine molecule. (**C**) Schematic of the use of SnoopLigase and sortase A in a two-step procedure of the production of dimeric dual-warhead FGF2 conjugate. (**D**) Structures of maleimide derivatives of α-amanitin (maleimide-αAMTN) and monomethyl auristatin E (maleimide-MMAE) used for conjugation to FGF2 variants. Both drugs contain a valine–citrulline p-aminobenzylcarbamate linker that is cleaved by intracellular proteases.

Based on our results, we have identified the N- to C-end FGF2 dimer coupled by a ~90 Å length flexible linker (N-PEG12-C dimer) as the most promising candidate for use as a drug carrier, due to its exceptional efficiency in promoting FGFR1-dependent cellular uptake [32]. For the purpose of the current study, we designed and produced in bacterial expression system a dimer whose structure closely resembled that of a chemical N-PEG12-C dimer. We opted to apply recombinant DNA technology in order to streamline the production process of the dimer as much as possible and to optimize its efficiency. The dimer developed in this study consisted of two FGF2 monomers’ variants. The first variant in the sequence had a C-terminal GGSKSK linker and C78S C96S point mutations, while the second variant contained an N-terminal KCKSGG linker and C78S C96S point mutations. To separate discrete FGF2 domains within the dimer, we used a flexible glycine-rich synthetic amino acid linker, (SGG)_11_ [34,35]. The number of repeating SGG motifs was determined so that the length of the spacer corresponded to the length of the chemical linker applied in the N-PEG12-C dimer. Additionally, we fused an N-terminal DIPATYEFTDGKHYITNEPIPPK and C-terminal LPETGG motifs to enable enzymatic attachment of cytotoxic drugs using SnoopLigase and evolved sortase A (eSrtA). We called the resulting FGF2 dimer dFGF2_V1V2_ (Figure 1C).

#### 2.1.2. α-AMTN and MMAE Conjugation Reaction Scheme

In the pursuit of developing a potent dimeric FGF2 dual-warhead conjugate, our payloads of choice were α-amanitin (α-AMTN) and monomethyl auristatin E (MMAE), two powerful drugs that exert diverse mechanisms of action [36]. α-AMTN is a toxic cyclic peptide derived from Amanita mushroom species. Its cytotoxic action stems from its selective binding to RNA polymerase II in eukaryotic cells, thereby inhibiting DNA transcription and leading to cell death. Moreover, due to its hydrophilic structure, it is not effectively removed by multi-drug resistant transporters [37,38]. This feature makes it potentially useful for targeting cancer cells that are resistant to other drugs. MMAE, on the other hand, is a synthetic analog of dolastatin 10, a natural anticancer compound. MMAE works by affecting tubulin polymerization, which is crucial for cell division. This disruption to cell division results in cell death, making MMAE a common payload in many antibody–drug conjugates (ADCs) [39,40,41]. The combination of α-amanitin and MMAE in a dual-warhead conjugate is anticipated to provide several advantages, including: (i) diverse mechanisms of action: the conjugate can target cancer cells through multiple pathways, increasing the likelihood of effectively killing them and overcoming resistance mechanisms [36]; (ii) synergistic effects: the combination of the two cytotoxic agents may produce a synergistic effect, where their combined action is greater than the sum of their individual effects, enhancing therapeutic efficacy [42]; (iii) overcoming drug resistance: the simultaneous targeting of cancer cells through different mechanisms can help circumvent drug resistance, as the cancer cells are less likely to develop resistance to both drugs at the same time [43].

Our conjugation strategy was based on direct enzymatic ligation. Enzymatic ligation is often considered a better approach than chemical conjugation in the context of drug to protein conjugation for several reasons. First, it can be highly specific, allowing for the selective modification of a single amino acid residue on a protein [44]. In contrast, chemical conjugation can result in non-specific modification of multiple sites, which can lead to decreased protein activity and/or stability [45]. Second, enzymatic ligation can be more efficient than chemical conjugation, as it can occur under mild conditions and does not require harsh chemicals or elevated temperatures that can damage proteins. This can result in higher yields and better preservation of protein activity [46,47]. Third, enzymatic ligation can be more versatile than chemical conjugation, as it can accommodate a wider range of drug molecules and protein targets [48].

**Figure 2:**
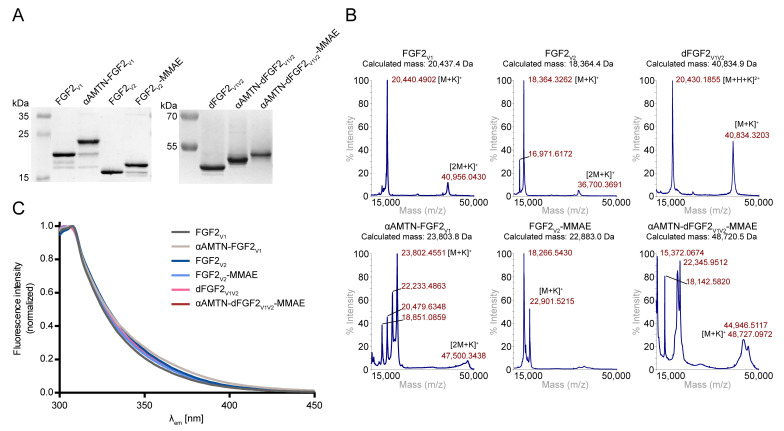
Conjugation of FGF2 variants with α-amanitin and monomethyl auristatin E. (**A**) Electrophoretic analysis of the products of conjugation reactions. (**B**) Mass spectra of FGF2 variants before (upper panel) and after (lower panel) the reaction. (**C**) Fluorescence emission spectra of FGF2 variants and their conjugates. Measurements were performed at a protein concentration of 4 × 10^−6^ M upon excitation at 280 nm. Curves were normalized to tyrosine emission at 303 nm.

We produced homogenous FGF2_V1_, FGF2_V2_, and dFGF2_V1V2_ and attached αAMTN and/or MMAE cytotoxic agents via SnoopLigase- or eSrtA-mediated ligation, respectively (Figure 1A–C and Figure 2A). Both drugs contained cathepsin B-cleavable Val-Cit dipeptide with para-aminobenzylcarbamate (PABC) selfimmolative spacer, which facilitated the release of free drug in the cell interior (Figure 1D) [49].

SnoopLigase is a recently discovered enzyme that catalyzes isopeptide bond formation between reactive lysine located on DIPATYEFTDGKHYITNEPIPPK and reactive asparagine on KLGSIEFIKVNK, in the presence of catalytic glutamic acid located on SnoopLigase. To enable SnoopLigase-mediated covalent conjugation of αAMTN to FGF2_V1_ and dFGF2_V1V2_, first, we synthesized NH_2_-KLGSIEFIKVNK-Ado_2_-C-CONH_2_ peptide (KLGSIEFIKVNK-PEG4-C). The SnoopLigase-recognized sequence KLGSIEFIKVNK was attached to cysteine through an Ado–Ado spacer to increase the hydrophilicity and to avoid steric conflicts between FGF2 and peptide–drug conjugate. Then, a maleimide derivative of α-amanitin (maleimide-αAMTN) (Figure 1D) was attached to KLGSIEFIKVNK-PEG4-C via a maleimide–thiol reaction. SnoopLigase efficiently ligated KLGSIEFIKVNK-PEG4-C-vcαAMTN peptide to DIPATYEFTDGKHYITNEPIPPK fusion proteins (FGF2_V1_, dFGF2_V1V2_), resulting in αAMTN-FGF2_V1_ and αAMTN-dFGF2_V1V2_ conjugates. The homogeneity and purity of the products was demonstrated by SDS-PAGE (Figure 2A).

For a site-specific conjugation of MMAE, we employed eSrtA (Figure 1B,C) that was catalytically optimized by a yeast display screening [50]. The eSrtA is a sequence-specific protease with a transpeptidase activity. The enzyme recognizes a LPXTG motif (X is typically any amino acid residue) and cleaves the threonine–glycine amide bond using the cysteine residue as a nucleophil. The resulting acyl–enzyme intermediate is aminolyzed by the attack of an incoming oligoglycine molecule, leading to the formation of a new peptide bond and fusion of the LPXT- and oligoglycine-linked peptides or proteins [51,52]. To conjugate MMAE to the FGF2_V2_ and αAMTN-FGF2_V1_ via eSrtA-mediated ligation, we synthesized NH_2_-GGGG-Ado_6_-C-CONH_2_ peptide (GGG-PEG12-C) and attached a maleimide derivative of monomethyl auristatin E (maleimide-MMAE) (Figure 1D) via maleimide–thiol chemistry. Next, we added excessive concentrations of GGG-PEG12-C-vcMMAE over FGF2_V2_ protein and αAMTN-dFGF2_V1V2_ conjugate to fully convert FGF2_V2_ to FGF2_V2_-MMAE and αAMTN-dFGF2_V1V2_ to αAMTN-dFGF2_V1V2_-MMAE, as demonstrated by SDS-PAGE (Figure 2A).

We confirmed the identity of FGF2_V1_, FGF2_V2_, dFGF2_V1V2_ proteins and their conjugates with αAMTN and/or MMAE with MALDI-MS (Figure 2B). Additional signals observed in the spectra arose from the fragmentation of the analyte molecules during the desorption/ionization process [53].

Moreover, to assess the proper folding of FGF2_V1_, FGF2_V2_, dFGF2_V1V2_ proteins and αAMTN-FGF2_V1_, FGF2_V2_-MMAE, αAMTN-dFGF2_V1V2_-MMAE conjugates, we utilized tryptophan fluorescence measurements. In native FGF2, tryptophan fluorescence is quenched by neighboring histidine and proline residues with tyrosine residues dominating the emission spectrum at a maximum of 303 nm. Upon unfolding, the quenching effect is diminished, resulting in a substantial increase in fluorescence at 353 nm. The fluorescence emission spectra indicate the folded native state of all investigated proteins and conjugates (Figure 2C).

### 2.2. Evaluation of FGFR1 Expression Levels and Accessibility for FGF2 in Various Cancer Cell Lines

**Figure 3:**
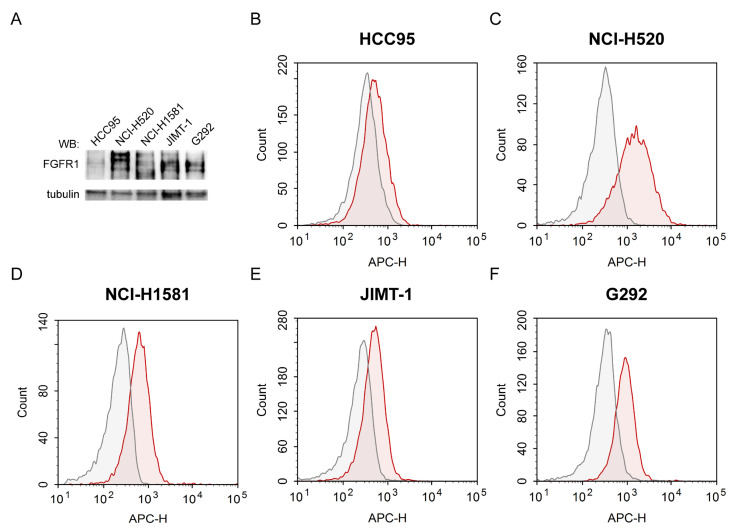
Determination of the FGFR1 level and its accessibility to FGF2. (**A**) Protein levels of FGFR1 in all cancer cell lines incorporated for the study, determined via Western blotting. The level of tubulin served as a loading control. (**B**–**F**) Flow cytometric analysis of FGF2 binding and cellular internalization. The cells were incubated on ice with 20 nM DyLight 650-labeled FGF2 for 40 min, then transferred to 37 °C for 30 min. Representative results of three independent experiments are shown.

To determine the cytotoxic potency and FGFR1 selectivity of the dimeric dual-warhead conjugate, we employed a panel of five cancer cell lines with varying FGFR1 levels. We utilized FGFR1-positive NCI-H520 and NCI-H1581 lung carcinomas, JIMT-1 breast ductal carcinoma, and G292 osteosarcoma. We also used HCC95, previously characterized as lung cancer cells with a low physiological FGFR1 levels [54,55,56]. The expression level of FGFR1 in all tested cell lines was analyzed with Western blotting (Figure 3A). Furthermore, the level of FGFRs accessible for FGF2 was indirectly assessed via flow cytometric measurements of the intensity of the fluorescent signal coming from DyLight 650-labeled FGF2 internalized to the cells or bound to the cell surface (Figure 3B–F and Table S1). The strongest signal from the fluorophore-tagged FGF2 was detected in NCI-H520 cells (Figure 3C), consistent with the high expression level of FGFR1 in these cells. NCI-H1581 (Figure 3D), JIMT-1 (Figure 3E) and G292 (Figure 3F) cells also accumulated significant or intermediate levels of FGF2. A detectable signal from the fluorescently labeled FGF2 was observed in HCC95 cells as well (Figure 3B), although it was significantly lower than in the other cell lines (Table S1). This finding aligns with previous reports indicating HCC95 is a cell line with low levels, but not devoid, of FGFR1 [54,57]. The data clearly suggest that FGF2 internalization primarily occurs through receptor-mediated endocytosis. Moreover, the different cell lines displayed distinct FGF2 internalization efficiencies, likely due to variations in FGFR1 levels on their surfaces.

### 2.3. Superior Cytotoxicity of the Dimeric Dual-Warhead Conjugate

The main aim of this study was evaluation of the cytotoxic potency of αAMTN-dFGF2_V1V2_-MMAE conjugate against cancer cell lines exhibiting overexpression of FGFR1. For this purpose, each cell line utilized in the experiment was treated with increasing concentrations of αAMTN-dFGF2_V1V2_-MMAE, nondimerized αAMTN-FGF2_V1_ and FGF2_V2_-MMAE conjugates mixed in equimolar ratio (αAMTN-FGF2_V1_ +FGF2_V2_-MMAE), αAMTN-FGF2_V1_, FGF2_V2_-MMAE, αAMTN, and MMAE. Cytotoxicity was assessed after 96 h of continuous exposure. The EC_50_ values were calculated from the Hill equation and are presented in Table 1.

**Figure 4:**
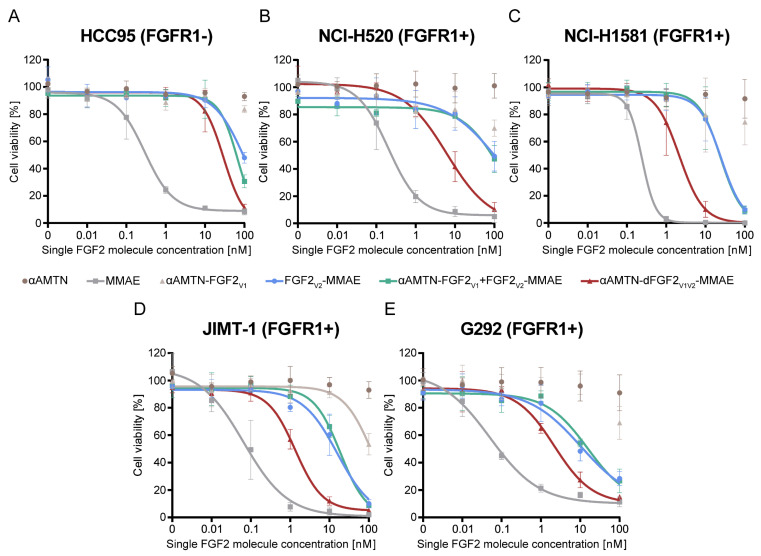
Cytotoxicity of the dimeric dual-warhead FGF2 conjugate against HCC95 control cell line with low physiological FGFR1 level (**A**) and FGFR1-overproducing cells (**B**–**E**). Viability of cells treated with the increasing concentrations of the αAMTN-dFGF2_V1V2_-MMAE, αAMTN-FGF2_V1_ and FGF2_V2_-MMAE conjugates applied individually or in combination therapy, as well as free drugs. The concentrations of all conjugates were normalized to the molar concentration of FGF2 molecules in the preparation. The presented data are mean values from three experiments ± SD. The solid lines represent Hill equation fits.

As shown in Figure 4, αAMTN-dFGF2_V1V2_-MMAE exhibited superior cytotoxicity against all investigated FGFR1-overexpressing cells, surpassing both monosubsituted αAMTN-FGF2_V1_ and FGF2_V2_-MMAE conjugates used in monotherapy, as well as combination therapy with equimolarly mixed αAMTN-FGF2_V1_ and FGF2_V2_-MMAE. In the case of HCC95, partial cytotoxicity was observed with FGF2_V2_-MMAE and αAMTN-dFGF2_V1V2_-MMAE at the highest tested concentration (Figure 4A). This phenomenon could potentially be attributed to the presence of low physiological levels of FGFR1 on the surface of HCC95 cells. Alternatively, there might be some target-independent toxicity of MMAE released from the conjugates. All tested conjugates exhibited cytotoxicity against FGFR1-positive cell lines: NCI-H520, NCI-H1581, JIMT-1 and G292 in a concentration-dependent manner. Interestingly, NCI-H520 cell line characterized by relatively high FGFR1 level (Figure 3A) and the most efficient cellular uptake of wild-type FGF2 (Figure 3B), was barely sensitive to single FGF2 conjugates with αAMTN or MMAE (including the mixture of both) in a studied concentration rage (Figure 4B). However, application of the αAMTN-dFGF2_V1V2_-MMAE dimeric dual-warhead conjugate led to a significant reduction in cell viability, with a decrease of over 90% observed at the highest tested concentration (Figure 4B). The calculated EC_50_ value of the dimeric conjugate was more than 10 times lower compared to equimolarly mixed αAMTN-FGF2_V1_ and FGF2_V2_-MMAE. Similar outcomes were observed in other cell lines with high FGFR1 expression levels. The EC_50_ values of αAMTN-dFGF2_V1V2_-MMAE were in the low nanomolar range and were at least one order of magnitude lower compared to the EC_50_ of mixed αAMTN-FGF2_V1_ and FGF2_V2_-MMAE, except for G292 cells, where, nonetheless, a large 5.8-fold reduction in EC_50_ was evident (Table 1, Figure 4C–E).

The EC_50_ values of αAMTN-dFGF2_V1V2_-MMAE and mixed single-drug FGF2 conjugates, calculated for the FGFR1-low HCC95 cell line, were comparable and significantly higher than the EC_50_ values calculated for other FGFR1-positive cell lines. A very low response observed in the FGFR1-deficient cell line indicates that the dimeric FGF2 dual-warhead conjugate exhibits excellent selectivity towards FGFR1-expressing cells.

In all studied cell lines, it has been observed that the conjugate of αAMTN was more toxic than free non-conjugated αAMTN, but the conjugate of MMAE was less toxic than free non-conjugated MMAE. The hydrophilic nature of αAMTN limits its efficient uptake by cancer cells, thereby reducing its ability to exert cytotoxic effects on the target cells [58,59]. In contrast, free MMAE is able to freely pass through cellular membranes, which allows it to exert its cytotoxic activity without limitations in cellular delivery, though it may lack selectivity [60,61]. However, when αAMTN and MMAE are conjugated to the FGF2 targeting protein, the conjugates utilize FGFR1-mediated endocytosis for cellular delivery, ensuring selectivity towards FGFR1-bearing cells. These findings highlight the efficiency of FGF2 as a targeting molecule in the conjugates.

### 2.4. Selective and Efficient Internalization of the αAMTN-dFGF2_V1V2_-MMAE Conjugate into Cells Expressing FGFR1

**Figure 5:**
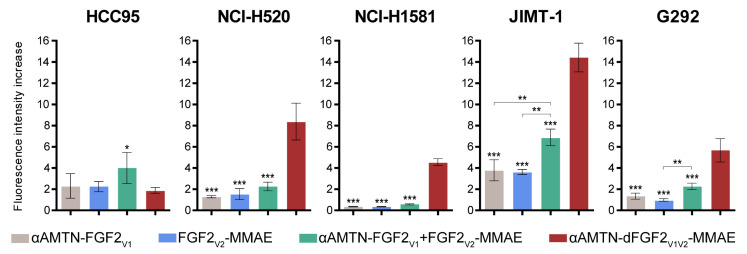
Internalization of the αAMTN-dFGF2_V1V2_-MMAE and αAMTN-FGF2_V1_ and FGF2_V2_-MMAE conjugates used separately or simultaneously in an equimolar ratio. For determination of internalization efficiency, different cancer cell lines were incubated for 15 min at 37 °C with Alexa Fluor 555-labeled conjugates at a concentration of 2 nM (concentrations were normalized to the molar concentration of FGF2 molecules in the preparation). Results represent the relative fluorescence intensities (∆I/I_0_) from three independent experiments. Values of the relative fluorescence intensities are the means for each data set ± SD. Statistical significance (versus αAMTN-dFGF2_V1V2_-MMAE group): * *p* < 0.05, ** *p* < 0.01, *** *p* < 0.001.

The success of cytotoxic conjugate-based anticancer therapy largely depends on the targeted delivery of toxic drugs, specifically to cancer cells. As we observed that the internalization capacity of wild-type FGF2 did not entirely correlate with the sensitivity of cell lines towards FGF2 conjugates, we further examined the internalization of all investigated conjugates using the same panel of cell lines that were used for cytotoxicity evaluation. To assess internalization efficiency, we labeled the FGF2 conjugates with a fluorescent probe and quantified their uptake using flow cytometry at a concentration close to the EC_50_ value of the dimeric conjugate (2 nM) (Figure 5). We aimed to determine if the shift in cytotoxicity towards lower concentrations was due to a higher internalization yield of the dimeric conjugate at low concentrations. To eliminate non-internalized fluorophore-labeled preparations from the cell surface, we incorporated acid washing as a step in our experimental protocol. Analysis of the internalization yield revealed that the αAMTN-dFGF2_V1V2_-MMAE dimeric conjugate exhibited significantly enhanced cell uptake efficiency compared to both the αAMTN-FGF2_V1_ and FGF2_V2_-MMAE monosubstituted conjugates, as well as the mixture of both, in the case of all FGFR1-overexpressing cell lines, but not in FGFR1-low HCC95 cells. The relative fluorescence intensity increase in HCC95 cells treated with αAMTN-dFGF2_V1V2_-MMAE was more than twice as low as that observed for cells treated with the combination of αAMTN-FGF2_V1_ + FGF2_V2_-MMAE. These data confirm that αAMTN-dFGF2_V1V2_-MMAE is an effective and selective conjugate for targeting FGFR1-positive cancer cells. Furthermore, the strong cytotoxicity displayed at low nanomolar concentrations aligns well with the internalization efficiency, substantiating the FGF2-mediated selective delivery mechanism responsible for the observed cytotoxic effects.

αAMTN-dFGF2_V1V2_-MMAE had a greater cytotoxic effect than any of the single-drug FGF2 conjugates, which could be explained as a result of the combined cytotoxic action of αAMTN and MMAE. Previously, we synthesized a bifunctional FGF2 conjugate, incorporating both αAMTN and MMAE as cytotoxic agents. These moieties were covalently linked to a cysteine residue through maleimide chemistry and propargyl lysine utilizing copper(I)-catalyzed azide-alkyne 1,3-dipolar cycloaddition (CuAAC), respectively. This dual-warhead conjugate exhibited enhanced cytotoxic efficacy against cancer cell lines with FGFR1 overexpression, surpassing the performance of single-drug conjugates [30]. We also produced a modular dual-warhead conjugate comprising two monosubsituted FGF2 molecules joined together via eSrtA-mediated ligation and demonstrated that conjugate containing both αAMTN and MMAE shows a greater cytotoxic effect than any of the single-drug FGF2 conjugates [31]. However, the dimerization method utilized previously requires a two-step enzymatic reaction compared to the bacterial production of the N-to-C dFGF2_V1V2_ dimer described here. Additionally, the previously utilized C-to-C dimer shows lower internalization efficacy compared to the N-to-C-end dFGF2_V1V2_ dimer. In our current study, we demonstrate that the utilization of a dimeric dual-warhead conjugate could be a highly promising approach, enhancing the efficacy of cancer therapies while minimizing potential side effects. This is attributed to the superior internalization capacity of the dimeric conjugate in cells with high FGFR1 levels, which leads to increased and selective cytotoxicity. Our findings suggest that the use of a dimeric conjugate may offer a more effective and targeted treatment strategy for cancers with high FGFR1 expression, highlighting its potential for improved therapeutic outcomes.

## 3. Materials and Methods

### 3.1. Reagents

All chemical reagents were procured from commercial sources and utilized as received, without additional purification. Reagents used for the solid-phase peptide synthesis are as follows. Fmoc-Cys(Trt) TentaGelS RAM resin (particle size: 90 μm, loading 0.2–0.25 mmol/g) purchased from Rapp Polymere GmbH (Tübingen, Germany). Amino acids: Fmoc-L-Lys(Boc)-OH, Fmoc-L-Asn(Trt)-OH, Fmoc-L-Val-OH, Fmoc-L-Ile-OH, Fmoc-L-Phe-OH, Fmoc-L-Glu(OtBu)-OH, Fmoc-L-Ser(tBu)-OH, Fmoc-Gly-OH, Fmoc-L-Leu-OH, Fmoc-O_2_Oc-O_2_Oc–OH piperidine, TIS (triisopropylsilane), DIPEA (*N*,*N*-diisopropylethylamine), DMF (*N*,*N*′-dimethylformamide), DCM (dichloromethane), and TFA (trifluoroacetic acid) from Iris Biotech GmbH (Marktredwitz, Germany). HPLC pure acetonitrile and Et_2_O (diethyl ether) were acquired from Avantor (Gliwice, Poland); FA (formic acid), TCA (trichloroacetic acid), and TFA (trifluoroacetic acid) from Merck (Darmstadt, Germany). TCE (2,2,2-trichloroethan-1-ol) wasfrom Santa Cruz Biotechnology (Dallas, TX, USA). DTNB (Ellman’s Reagent) (5,5-dithio-bis-(2-nitrobenzoic acid)) was from Thermo Fisher Scientific (Waltham, MA, USA). The toxins: α-amanitin and MC-vc-PAB-C_6_-α-amanitin were from Levena Biopharma Co., Ltd. (San Diego, CA, USA). MMAE (monomethyl auristatin E) and MC-vc-PAB−MMAE were from MedChemExpress (Monmouth Junction, NJ, USA). Alexa Fluor 555 C_2_ Maleimide and DyLight 650 NHS Ester dyes, and alamarBlue reagent were from Thermo Fisher Scientific. The chromatographic columns: HiTrap Desalting with Sephadex G-25 resin, HiTrap Heparin HP and HisTrap HP with Ni-Sepharose media were from GE Healthcare (Chicago, IL, USA). Zeba Spin Desalting columns were from Thermo Fisher Scientific. Synergi 4 μm Fusion-RP 80 Å 250 × 10 mm^2^ LC column was from Phenomenex Inc. (Torrance, CA, USA). All other reagents were purchased from Sigma-Aldrich (Saint Louis, MO, USA) or BioShop Canada Inc. (Burlington, ON, USA).

### 3.2. Cell Lines

The NCI-H520 (human squamous cell carcinoma, HTB-182), NCI-H1581 (human non-small cell lung cancer, CRL-5878), and G292 clone A141B1 (human osteosarcoma, CRL-1423) cell lines were obtained from American Type Culture Collection (ATCC, Manassas, VA, USA); JIMT-1 (human breast ductal carcinoma) cells were acquired from the Leibniz Institute DSMZ-German Collection of Microorganisms and Cell Cultures (Braunschweig, Germany); whereas HCC95 cell lines derived from human lung squamous carcinoma were procured from Sigma-Aldrich. NCI-H520 cells were cultured in RPMI 1640 medium from ATCC; NCI-H1581 in RPMI 1640 from BioWest (Nuaillé, France); G292 and JIMT-1 in DMEM High Glucose medium with stable glutamine and sodium pyruvate (BioWest); and HCC95 in RPMI 1640 (ATCC) with the addition of 0.1% sodium bicarbonate (Gibco, Billings, MT, USA). All culture media were supplemented with 10% fetal bovine serum from Thermo Fisher Scientific, and 1% penicillin/streptomycin mix from BioWest. All cell lines were maintained in a humidified incubator at 37 °C in 5% CO_2_ atmosphere and were seeded onto tissue culture plates the day before initiating the experiments.

The cell lines used in the study were examined for the presence of the FGFR1 via Western blotting. The cells were lysed in 4× SDS-PAGE loading buffer (200 mM Tris-HCl pH 6.8, 8% SDS, 0.4% bromophenol blue, 50 mM TCEP, 40% glycerol), sonicated, and boiled. Cell lysates were resolved by 10% SDS-PAGE, transferred to the Immobilon-PSQ PVDF 0.2 μm membrane (Millipore, Darmstadt, Germany) and immunoblotted with the primary antibody directed against FGFR1 (#9740) purchased from Cell Signaling (Danvers, MA, USA). Tubulin served as a loading control and was detected with the antibody (#T6557) from Sigma-Aldrich. HRP-conjugated anti-mouse (115-035-003) and anti-rabbit (111-035-144) secondary antibodies were obtained from Jackson Immuno-Research Laboratories (Cambridge, UK).

### 3.3. Recombinant Proteins

For the purpose of this work, three FGF2 variants were used: (a) FGF2 with N-terminal DIPATYEFTDGKHYITNEPIPPK, C-terminal GGSKCK motifs and two point mutations (C78S, C96S) (FGF2_V1_), (b) FGF2 with N-terminal KCKSGG, C-terminal LPETGG motifs and two point mutations (C78S, C96S) (FGF2_V2_), and (c) dimer consisting of FGF2_V1_ and FGF2_V2_ moieties joined by (SGG)_11_ linker (dFGF2_V1V2_). The pET-3a and pET-11d plasmids carrying cDNA of FGF2_V1_ and dFGF2_V1V2_, respectively, were purchased from the Gene Universal (Newark, DE, USA) as a custom gene synthesis. The LPETGG sequence was introduced to the FGF2 with N-terminal KCKSGG linker and C78S C96S point mutations via site-directed mutagenesis [31]. All FGF2 proteins were produced in *Escherichia coli* (*E. coli*) Rosetta 2(DE3)pLysS (Novagen-EMD Biosciences, Madison, WI, USA). Individual colonies of transformed *E. coli* were grown in LB medium with 100 µg/mL ampicilin and 30 µg/mL chloramphenicol for 16 h at 37 °C, 200 rpm. Overnight cultures were then diluted 1:50 in a TB medium with 100 µg/mL ampicillin and cultivated at 37 °C, 200 rpm to OD_600_ 2.0. The protein expression was induced by the addition of IPTG to a final concentration of 0.5 mM. The production of FGF2_V1_ was continued for 1h under the same conditions. In turn, cultures of FGF2_V2_ and dFGF2_V1V2_ were incubated for 16 h at 25 °C (FGF2_V2_) or 16 °C (dFGF2_V1V2_), 200 rpm. The bacteria were harvested via centrifugation at 7500× *g*, resuspended in 50 mM monosodium phosphate pH 7.4, 0.15 M NaCl, 2 mM DTT, 1 mM EDTA, 0.1% Triton X-100, 1 mM PMSF, 250U Universal Nuclease (Thermo Fisher Scientific), and homogenized via sonication on ice. The cell lysates were centrifuged at 50,000× *g* at 4 °C for 1 h. The clarified supernatants were diluted with elution buffer (50 mM monosodium phosphate pH 7.4 with 2 M NaCl, 2 mM DTT, 1 mM EDTA, 1 mM NH_4_SO_4_) to a final NaCl concentration of 0.5 M. FGF2 proteins were purified via affinity chromatography using a HiTrap Heparin HP column and NGC chromatography system (Bio-Rad, Hercules, CA, USA). Proteins were eluted from the column with a linear 0.5–2 M gradient of NaCl, except for dFGF2_V1V2_, where 0.5–3 M gradient was applied.

The pET28a-AviTag-SnoopLigase was a gift from Mark Howarth (Addgene plasmid #105626) [46]. The expression plasmid was transformed into *E. coli* Rosetta 2(DE3)pLysS (Novagen-EMD Biosciences). A single colony of transformed *E. coli* was inoculated into LB medium with 50 µg/mL kanamycin and 30 µg/mL chloramphenicol and incubated for 16 h at 37 °C, 200 rpm. Starter cultures were then diluted 1:50 in TB medium with 50 µg/mL kanamycin and grown at 37 °C, 200 rpm until OD_600_ 2.0. Protein expression was induced with 0.42 mM IPTG and protein production was carried out overnight at 30 °C, 200 rpm. SnoopLigase was purified using HisTrap HP column with Ni-Sepharose media. Chromatography was performed on the NGC system (Bio-Rad). The procedure was performed according to the standard protocol for purification of histidine-tagged proteins (GE Healthcare). SnoopLigase was eluted with a linear 10–500 mM gradient of imidazole. Following the elution, protein fraction was desalted using a HiTrap Desalting column with Sephadex G-25 resin. SnoopLigasse was stored in TB pH 7.6 (50 mM Tris base adjusted to pH 7.6 with boric acid) with 5% glycerol.

The evolved sortase A pentamutant (eSrtA) in pET29 was a gift from David Liu (Addgene plasmid #75144) [50] and was produced and purified as already described [31].

### 3.4. Synthesis and Purification of KLGSIEFIKVNK-PEG4-C-vcαAMTN and GGGG-PEG12-C-vcMMAE

Synthesis of NH_2_-KLGSIEFIKVNK-Ado_2_-C-CONH_2_ and NH_2_-GGGG-Ado_6_-C-CONH_2_ was performed on solid support according to the solid phase peptide synthesis (SPPS) method described as part of the Fmoc strategy. The peptides were cleaved from the resin with a mixture of TFA/DMC/TIS/H_2_O (vol%, 95:3:1:1) or TFA/DMC/EDT/TIS/H_2_O (vol%, 93:3:2:1:1), respectively. The crude peptides were triply precipitated in cold Et_2_O, purified by reversed-phase high-performance liquid chromatography (RP-HPLC), and lyophilized. Both samples were dissolved in *N*,*N*-dimethylacetamide (DMAc) and peptides’ concentration was estimated by assaying sulfhydryl groups with Ellman’s reagent (Thermo Fisher Scientific).

Conjugation of NH_2_-KLGSIEFIKVNK-Ado_2_-C-CONH_2_ (1.5 µmol) with MC-vc-PAB-C6-α-amanitin (1.0 µmol) was performed in DMAc (275 µL) in the presence of DIPEA (10 µmol). The reaction was carried out for 1 h at room temperature on the rotator with gentle shaking. MC-vc-PAB−MMAE (5 µmol) was conjugated to NH_2_-GGGG-Ado_6_-C-CONH_2_ (10 µmol) in 500 µL DMAc with the addition of DIPEA (50 µmol). The reaction was conducted at room temperature for 6 h. The peptide–drug conjugates were purified from the reaction mixtures using RP-HPLC and lyophilized. The identity of the products was confirmed by MALDI-MS.

### 3.5. Synthesis of the Conjugates

#### 3.5.1. Conjugation of the FGF2_V1_ and dFGF2_V1V2_ with αAMTN via SnoopLigase-Mediated Ligation

FGF2_V1_ and dFGF2_V1V2_ proteins containing N-terminal DIPATYEFTDGKHYITNEPIPPK motif were monosubstituted with KLGSIEFIKVNK-PEG4-C-vcαAMTN peptide–drug conjugate in the reaction catalyzed by SnoopLigase. FGF2_V1_ and dFGF2_V1V2_ were transferred into the reaction buffer (25 mM HEPES pH 7.6, 15% glycerol, 2 mM TCEP) using HiTrap Desalting column with Sephadex G-25 resin. KLGSIEFIKVNK-PEG4-C-vcαAMTN was dissolved in DMAc to prepare 10 mM stock solution. Next, 10 µM FGF2_V1_ or dFGF2_V1V2_ was incubated with 80 µM KLGSIEFIKVNK-PEG4-C-vcαAMTN and 30 µM SnoopLigase for 24 h at 15 °C. The reaction mixture was then loaded onto a HiTrap Heparin HP column. The excess of nonreacted and nonimmobilized peptide and protein was immediately removed by washing the resin with reaction buffer. However, upon reaction, SnoopLigase strongly binds to the reaction product. To disrupt SnoopLigase-reaction product interaction and allow efficient purification of the ligated product, the column was washed with 3 resin volumes of 2 M imidazole pH 7.0, then washed with 5 resin volumes of 25 mM HEPES pH 7.6, 0.154 M NaCl. The resulting αAMTN-FGF2_V1_ or αAMTN-dFGF2_V1V2_ conjugate, free from SnoopLigase, was eluted from the column with 25 mM HEPES pH 7.6, 2.5 M NaCl, 10 mM Na_2_SO_4_, 5 mM CaCl_2_.

#### 3.5.2. Double-Warhead Conjugate Synthesis. Conjugation of the αAMTN-dFGF2_V1V2_ and FGF2_V2_ with MMAE via eSrtA-Mediated Ligation

GGGG-PEG12-C-vcMMAE peptide–drug conjugate was coupled with αAMTN-dFGF2_V1V2_ conjugate and FGF2_V2_ protein containing C-terminal LPETGG sequence using eSrtA. For the reaction, αAMTN-dFGF2_V1V2_ and FGF2_V2_ were buffered to 25 mM HEPES pH 7.6, 154 mM NaCl, 10 mM Na_2_SO_4_, 5 mM CaCl_2_, and 2 mM TCEP using a HiTrap Desalting column with Sephadex G-25 resin. The reaction of 40 µM αAMTN-dFGF2_V1V2_ or FGF2_V2_ with 100 µM GGGG-PEG12-C-vcMMAE was carried out in the presence of 0.25 µM eSrtA for 4 h at 15 °C. To remove the catalyst and excess reactant, the reaction mixture was passed through a HiTrap Heparin HP column. Unbound molecules were washed off the column with 25 mM HEPES pH 7.6 and the target product of the reaction (αAMTN-dFGF2_V1V2_-MMAE or FGF2_V2_-MMAE) was eluted with 25 mM HEPES pH 7.6, 2.5 M NaCl, 2 mM TCEP. The efficiency of the conjugation was confirmed by SDS-PAGE. The identity of the conjugates was confirmed by MALDI-MS.

### 3.6. Mass Spectrometry

Molecular masses of peptides, proteins and their conjugates were verified by MALDI-TOF/TOF MS (Applied Biosystems AB 4800+, Waltham, MA, USA). α-cyano-4-hydroxycinnamic acid or sinapic acid were used as a matrix.

### 3.7. Spectrofluorimetry

Spectrofluorimetric analysis was carried out to determine the folding state of the FGF2 variants and their conjugates. The tryptophan fluorescence spectra were acquired using a FP-750 spectrofluorimeter (Jasco, Tokyo, Japan) with excitation at 280 nm and emission in the 300−450 nm range. Measurements were performed at 20 °C at ~4 µM sample concentration in PBS.

### 3.8. Cytotoxicity Assay

The cytotoxicity of the conjugates and free drugs was evaluated on NCI-H520, NCI-H1581, JIMT-1 and G292 cell lines characterized as FGFR1 amplified, as well as on HCC95 with low levels of FGFR1 [54]. Cells were seeded in 96-well plates at a density of 5 × 10^5^ cells/well in 100 µL of dedicated culture media 24 h prior to commencing the experiment. The media were then replaced with fresh complete media and the cells were treated with six concentrations of conjugate or free drug (0.01 to 100 nM range). The effect of the investigated preparations on cell survival was determined after 96 h of continuous exposure. Cell viability was measured using an alamarBlue reagent, according to the protocol provided by the manufacturer. Fluorescence emission of the reduced form of the reagent at 590 nm, upon excitation at 560 nm, was measured using an Infinite M1000 PRO microplate reader (Tecan, Mannedorf, Switzerland). Cytotoxicity experiments were performed in triplicate. To determine the half maximal effective concentration (EC_50_), the data were fitted to the Hill equation using the GraphPad Prism version 6.01 (GraphPad Software, San Diego, CA, USA). Statistical significance was determined using the paired Student’s t-tests assuming equal variance.

### 3.9. Labeling of Conjugates and Wild-Type FGF2 with Fluorescent Probes

Unmodified wild-type FGF2 (FGF2 WT) was labeled with DyLight 650 Succinimidyl Ester; in turn, αAMTN-FGF2_V1_, FGF2_V2_-MMAE and αAMTN-dFGF2_V1V2_-MMAE conjugates with Alexa Fluor 555 C_2_ Maleimide. For the labeling reaction, proteins were dissolved at 30 µM in a suitable buffer—FGF2 WT in 25 mM HEPES pH 8.0, 10 mM Na_2_SO_4_, conjugates in 25 mM HEPES pH 7.4, 10 mM Na_2_SO_4_, and 1 mM TCEP. Fluorescent probes were reconstituted in DMAc to prepare 10 µM stock solutions and were added directly tothe proper protein or conjugate solution in a 10-fold molar excess. The reaction was carried out at room temperature for 1 h in the dark. Non-reactive excess fluor reagent was removed from the protein using ZebaSpin Desalting columns (Thermo Fisher Scientific).

### 3.10. Flow Cytometric Evaluation of FGF2 Capacity to Bind to the Cells

The capacity of FGF2 to bind and to enter to the cells differing in total FGFR1 levels was analyzed as we reported earlier, with minor modifications [31]. HCC95, NCI-H520, NCI-H1581, JIMT-1, and G292 cells were seeded at a density of 5 × 10^5^ cells/well in a 6-well plate in complete culture medium and left overnight to adhere. The media were then replaced with serum-free formulations with 1% BSA and 10 U/mL heparin, and the cells were starved for 4 h. DyLight 650-labeled FGF2 WT was added directly to the culture media in a concentration of 20 nM. The cells were preincubated with the protein for 40 min on ice, and then transferred to 37 °C for another 30 min. The cells were then detached from the culture plate with 5 mM EDTA-PBS and subjected for flow cytometric analysis using a NovoCyte 2060R instrument (ACEA Biosciences, San Diego, CA, USA) and NovoExpress software (version 1.6.1) (ACEA Biosciences).

### 3.11. Flow Cytometric Analysis of Conjugates’ Steady-State Internalization

The method used for flow cytometric estimation of ligand internalization efficiency was adapted from the literature [62]. To determine the internalization yield, serum-starved HCC95, NCI-H520, NCI-H1581, JIMT-1, and G292 cells were treated with Alexa Fluor 555-labeled αAMTN-FGF2_V1_, FGF2_V2_-MMAE, and αAMTN-dFGF2_V1V2_-MMAE conjugates at a concentration of 2 nM, except for the group, where both αAMTN-FGF2_V1_ and FGF2_V2_-MMAE conjugates were added, at 1 nM concentration each. Incubation was carried out for 15 min at 37 °C. The cells were then rapidly placed on ice and extensively washed with ice-cold PBS in order to stop the receptor trafficking. Non-internalized conjugates were removed from the cell surface via multiple washes in acid stripping buffer (serum-free medium supplemented with 0.2% BSA, pH = 3.5). The cells were harvested with 5 mM EDTA-PBS and analyzed with a NovoCyte 2060R flow cytometer and NovoExpress software.

## 4. Conclusions

In summary, we developed a novel strategy for generating a dual-warhead cytotoxic conjugate through site-specific conjugation of cytotoxic payloads via SnoopLigase- and eSrtA-mediated ligation. Within this study, we explored αAMTN, a selective and efficacious RNA polymerase II and III inhibitor derived from Amanita mushrooms, as well as a highly potent antimitotic agent, an analog of auristatin (MMAE), commonly used in antibody–drug conjugates (ADCs). Our results confirm that the FGF2 dimer with a specific well-defined topology can serve as an effective drug delivery vehicle for targeting cancer cells that overproduce FGFR1. Specifically, the αAMTN-dFGF2_V1V2_-MMAE conjugate effectively kills cancer cells with high levels of FGFR1 while exhibiting minimal toxicity towards FGFR1-deficient cells. Our study successfully combines two distinct cytotoxic agents with the selective targeting properties of the FGF2 dimer, resulting in a highly potent and unique conjugate that has been validated in cellular models. The future research on the αAMTN-dFGF2_V1V2_-MMAE dimeric dual-warhead conjugate should prioritize in vivo evaluation in murine models to comprehensively assess their therapeutic potential. These studies are necessary not only to confirm the anti-cancer efficacy of our molecule, but also to assess its systemic cytotoxicity. Importantly, we previously developed a monomeric FGF2-based conjugate that exhibited high toxicity against FGFR1-overproducing cancer cells in vitro and showed efficient tumor growth retardation in an FGFR-positive human breast cancer xenograft model in mice [63]. Administration of this FGF2-based conjugate did not cause any body weight loss or other side effects in the animals during the treatment period. These data are very promising in the context of utilizing cytotoxic drug conjugates based on FGF2 in FGFR-targeted anti-cancer therapies.

## Figures and Tables

**Table 1:** Comparison of the cytotoxic effects of conjugates and free drugs on different cell lines with varying levels of FGFR1.

Preparation	Cell Line				
	HCC95	NCI-H520	NCI-H1581	JIMT-1	G292
	EC_50_ [nM]				
Non-conjugated αAMTN	1238 **	n.t. ^a^	998.6 **	1278 **	967.1 **
Non-conjugated MMAE	0.3398 **	0.2761 **	0.2288 **	0.09179 *	0.09103 **
αAMTN-FGF2_V1_	487.7 ***	205.5 ***	233.2 **	112.5 **	225.6 **
FGF2_V2_-MMAE	91.53 **	82.67 ***	22.60 **	12.83 *	10.77 *
αAMTN-FGF2_V1_ + FGF2_V2_-MMAE	52.29	73.46 ***	23.28 ***	16.40 **	14.45 **
αAMTN-FGF2_V1V2_-MMAE	**32.45**	**6.803**	**2.195**	**1.304**	**2.510**

^a^ n.t., Nontoxic in the studied concentration range; *t* test of three independent experiments was performed for statistical analysis of data. Statistical significance (versus αAMTN-FGF2_V1V2_-MMAE group): * *p* < 0.05, ** *p* < 0.01, *** *p* < 0.001. Bold indicate the molecule that was developed and described.

## Data Availability

Data will be made available on request.

## Supplementary Materials

The following supporting information can be downloaded at: https://www.mdpi.com/article/10.3390/ijms241210143/s1.

## Abbreviations

ADC	Antibody–drug conjugate.
ADCC	Antibody-dependent cellular cytotoxicity.
Ado	8-amino-3,6-dioxaoctanoyl.
ATCC	American Type Culture Collection.
BSA	Bovine serum albumin.
CDC	Complement-dependent cytotoxicity.
DCM	Dichloromethane.
DIPEA	*N*,*N*-diisopropylethylamine.
DMAc	*N*,*N*-dimethylacetamide.
DMF	*N*,*N*′-dimethylformamide.
DTNB	5,5-dithio-bis-(2-nitrobenzoic acid).
DTT	Dithiothreitol.
EC	Effective concentration.
EDTA	Ethylenediaminetetraacetic acid.
eSrtA	Evolved sortase A.
FA	Formic acid.
FGF	Fibroblast growth factor.
FGFR	Fibroblast growth factor receptor.
HEPES	4-(2-hydroxyethyl)-1-piperazineethanesulfonic acid.
HPLC	High-performance liquid chromatography.
HRP	Horseradish peroxidase.
IPTG	Isopropyl β-D-1-thiogalactopyranoside.
MALDI	Matrix-assisted laser desorption/ionization.
MMAE	Monomethyl auristatin E.
MS	Mass spectrometry.
TCEP	Tris(2-carboxyethyl)phosphine.
OD	Optical density.
PABC	Para-aminobenzylcarbamate.
PBS	Phosphate-buffered saline.
PEG	Poly(ethylene glycol).
PMSF	Phenylmethylsulfonyl fluoride.
PVDF	Polyvinylidene difluoride.
RP	Reversed phase.
SD	Standard deviation.
SDS-PAGE	Sodium dodecyl sulfate polyacrylamide gel electrophoresis.
SPPS	Solid phase peptide synthesis.
TB	Terrific broth.
TCA	Trichloroacetic acid.
TCE	2,2,2-trichloroethan-1-ol.
TFA	Trifluoroacetic acid.
TIS	Triisopropylsilane.
TOF	Time of flight.
WT	Wild-type.
αAMTN	α-amanitin.
