# Supplementary material for: Dual-Warhead Conjugate Based on Fibroblast Growth Factor 2 Dimer Loaded with α-Amanitin and Monomethyl Auristatin E Exhibits Superior Cytotoxicity towards Cancer Cells Overproducing Fibroblast Growth Factor Receptor 1
Source: Int J Mol Sci. 2023 Jun 14;24(12):10143. doi: 10.3390/ijms241210143 (PMC10299504; doi:10.3390/ijms241210143)
Supplement: Supplementary file 1 [file ijms-24-10143-s001.zip › ijms-2421467-supplementary.pdf]

International Journal of
Molecular Sciences

MDPI

Supplementary Materials

Dual-Warhead Conjugate Based on Fibroblast Growth Factor 2 Dimer Loaded with α-Amanitin and Monomethyl Auristatin E Exhibits Superior Cytotoxicity Towards Cancer Cells Overproducing Fibroblast Growth Factor Receptor 1

Daria Nawrocka, Mateusz Adam Krzyysscik †, Katarzyna Dominika Sluzalska and JaaccekOtlewski *

Department of Protein Engineering, Faculty of Biotechnology, University of Wroclaw, Joliot-Curie 14a, 50-383 Wroclaw, Poland

* Correspondence: jacek.otlewski@uwr.edu.pl; Tel.: +48-71-375-28-24
† Current address: Institute for NanoBioTechnology, Whiting School of Engineering, Johns Hopkins University, 3400 N Charles St, Baltimore MD 21218

Table S1. Flow cytometric analysis of FGF2 binding and cellular internalization. The cells were incubated on ice with 20 nM DyLight 650-labeled FGF2 for 40 min, then transferred to 37 °C for 30 min. Relative intensity change was normalized to the non-treated cells. Results represent the relative fluorescence intensities ($\Delta I/I_0$) from three independent experiments. Values of the relative fluorescence intensities are the means for each data set ± SD. Statistical significance (versus HCC95 cells): ** $p < 0.01$, ***$p < 0.001$.

Cell line	Relative intensity change ($\Delta I/I_0$)
HCC95	0.6513 ± 0.06759
NCI-H520	4.359 ± 0.7984***
NCI-H1581	2.604 ± 0.2655***
G292	1.506 ± 0.08039***
JIMT-1	1.079 ± 0.1031**
